# Role of four-week resistance exercise in preserving the heart against ischaemia–reperfusion-induced injury

**DOI:** 10.5830/CVJA-2012-050

**Published:** 2012-09

**Authors:** Yousef Doustar, Farhad G Soufi, Afshar Jafary, Mohaddeseh M Saber, Rafigheh Ghiassie

**Affiliations:** Department of Pathology, Faculty of Veterinary Medicine, Islamic Azad University (Tabriz branch), Tabriz, Iran; Drug Applied Research Centre, Tabriz University of Medical Sciences and Department of Physiology, Faculty of Medicine, Tabriz University of Medical Sciences, Tabriz, Iran; Department of Exercise Physiology, Faculty of Physical Education, Tabriz University, Tabriz, Iran; Drug Applied Research Centre, Tabriz University of Medical Sciences, Tabriz, Iran; Drug Applied Research Centre, Tabriz University of Medical Sciences, Tabriz, Iran

**Keywords:** exercise, heart, infarction, ischaemia, reperfusion

## Abstract

**Objective:**

We studied the cardioprotective effect of resistance training against ischaemia–reperfusion-induced injury.

**Methods:**

Forty male rats were divided into trained and sedentary groups (*n* = 20 for each). The trained rats were exercised at 12 repetitions/set, four sets/day and five days/week for four weeks. Transient regional ischaemia of the left anterior descending coronary artery (40 min) was followed by 80 min of reperfusion.

**Results:**

Baseline developed and diastolic pressures and coronary flow were similar in the two groups. While diastolic pressure increased and developed pressure and coronary flow decreased in both the ischaemic and perfusion periods (as indices of cardiac damage), there were no statistically significant differences between the trained and sedentary groups in these parameters. Resistance training did not significantly change the infarct size and apoptosis rate.

**Conclusion:**

We did not see a cardioprotective effect of resistance exercise against ischaemia–reperfusion-induced injury in this study. A precise conclusion about this issue needs more investigations.

## Abstract

Ischaemic heart disease remains a worldwide problem affecting all economic groups of society.^1^ The primary pathological manifestation of ischaemic heart disease is myocardial infarction due to ischemia–reperfusion (IR) injury.^2^ Preservation of cardiac performance and reduction of infarct size are the main goals in the management of IR-induced complications.^2^ In this regard, many approaches to providing cardioprotection against IR-induced injury have been studied.

Until now, regular exercise has been confirmed as a pragmatic and sustainable countermeasure for cardioprotection.^3^ While convincing evidence indicates that both short-term (three to five consecutive days) and long-term (months) endurance exercise training (i.e. running and swimming) improves myocardial tolerance to IR-induced injury in both male and female animals as well as young and old animals,^3^ there is no clear understanding of the cardioprotective effect of resistance exercise training (such as body building and weight lifting) against IR-induced injury.

Resistance exercise training is a specialised method of conditioning designed to increase strength and muscle endurance.^4^ Similar to endurance training, it has been shown that resistance training has beneficial effects on some physiological and pathological processes such as physical fitness, quality of life and chronic heart failure.^5^ While the risk of cardiovascular complications is the primary concern with resistance training in some cardiac patients (due to blood pressure elevation during this type of exercise), resistance training can positively influence quality of life, cardiovascular risk factors and cardiovascular function in healthy persons and in selected patients with cardiovascular disease.^5^,^6^

Although several investigators have studied the impact of resistance training on cardiac structure and function, the cardioprotective effect of resistance exercise training against IR-induced injury has not been understood. The purpose of this study was to investigate cardiac performance during the ischaemic and reperfusion periods, as well as to determine cardiac infarct size and apoptosis rate after IR-induced injury in rats undergoing resistance exercise training for a short period of four weeks.

## Methods

Forty male Wistar rats (220–240 g, three months old) were obtained from the laboratory animal house of Tabriz University of Medical Sciences and they were randomly divided into trained (EXT) and sedentary (Sed) groups (*n* = 20 for each group). Animals were housed at room temperature (23 ± 1°C) with 12-hour light/dark cycles and had free access to food and water. The study protocol was designed in accordance with the *Guide for the Care and Use of Laboratory Animals* published by the US National Institutes of Health (NIH publication, revised 1996) and approved by the Ethics Committee for the Use of Animals in Research of the Tabriz University of Medical Sciences.

Trained rats were exercised according to the model described by Tamaki *et al.*, with some modifications.^7^ Rats were placed vertically in a squat-training apparatus cylinder (RatWLI009, Tajhiz Azmaye Pooya Co, Iran) as they could stand on their hind limbs in response to electrical stimulation and raise the piston which was located above their heads. An electrical stimulation (20 V, 0.3-s duration at 3-s intervals) was applied to the rat’s tail through a surface electrode. After one week of adaptation, the trained group of rats exercised for four sets of 12 repetitions per day, with a 90-s rest period between each set, five times per week for four weeks.^4^

Each rat in the trained group was weighed daily and 120% of its body weight (approximately 70% of the maximum load that the rats were able to raise following electrical stimulation) was used to determine the weight of the piston. The piston movement for each rat was recorded by a distance sensor which had been located above the piston and the work performed by each rat was calculated daily by multiplying the piston weight and piston movement.

According to the method of Brown *et al.*, after anaesthetisation with pentobarbital sodium (35 mg/kg ip injection) the hearts were excised, placed in ice-cold saline and rapidly hung by the aorta on the cannula of the Langendorff apparatus.^8^ Hearts were perfused with 37.5°C Krebs buffer (76.5 mmHg perfusion pressure with 95% O_2_ and 5% CO_2_) containing 117.4 mM NaCl, 4.7 mM KCl, 1.9 mM CaCl_2_, 1.2 mM MgSO_4_, 1.2 mM KH_2_PO_4_, 5 mM pyruvate, 11 mM glucose, 0.5 mM EDTA, 25 mM NaHCO_3_ and 1200 U/l heparin.

A pressure-transducing catheter was placed through the cannula and aortic valve into the chamber of the left ventricle (LV) and the developed pressure was determined with a computer connected to the transducer (PowerLab, AD Instruments, Australia). After a 5-min stabilisation period, baseline pressure was measured, and coronary flow rate was obtained by collection of the coronary effluent for 1 min.

After baseline records, a suture was threaded through the left anterior descending coronary artery 3–5 mm distal to the aorta in 14 rats in each group. Both ends of the suture were inserted into a small polyethylene tube that was used as a snare, and ischaemia was induced by tightening the snare so that the artery was fully compressed. Pressure and coronary flow measurements were recorded at 5, 15, and 30 min after the onset of ischaemia. After 40 min, the snare was loosened and reperfusion ensued for 80 min. Coronary flow and pressure data were recorded at 5 min after the onset of reperfusion and then every 15 min until the end of the 80-min reperfusion period.

Data were omitted from analysis if the coronary flow did not decrease at the onset of ischaemia or increase at the onset of reperfusion (*n* = 3), or if the hearts did not complete the IR protocol due to fibrillation or technical difficulty (*n* = 2). Only 11 hearts in the control group and 12 in the trained group completed the IR protocol.

In the remaining rats from each group (*n* = 6), the hearts were excised, cannulated and perfused as described above but without the ischaemic period, to observe how the mechanical and flow measurements changed as a function of time. Pressure and flow were recorded in these hearts at the same time points as in the hearts that experienced ischaemia–reperfusion.

Infarct size was measured using methods similar to those previously described.^8^,^9^ After the reperfusion period, the snare was re-tightened around the left anterior descending coronary artery in six hearts from each group, and 100 μl of 0.05% Evans blue solution was injected into the aortic cannula and perfused through the heart for 3 min. Then the heart was sliced transversely from base to apex into four slices of equal width. Each slice was immersed in phosphate buffer and was photographed with a digital camera.

After both sides of each slice were photographed, each slice was placed in 100 mM phosphate buffer with 0.1% triphenyltetrazolium chloride and incubated for 10 min at 37°C. After incubation, each side of every slice was again photographed and the slices were weighed. Heart weight was obtained by summation of the slice weights for each heart.

To avoid experimenter bias, images of the slices were analysed in a single-blind manner by Scion Image 4.0 software. Total slice area (TA), zone at risk (ZAR: the area of each slice that did not turn blue after perfusion with the solution containing Evans blue dye) and infarct area (IA: the portion of the ZAR that did not turn red in response to triphenyltetrazolium chloride incubation and remained white) were measured. ZAR and IA were obtained from each side of a single slice, and the mean of both sides was used as the representative ZAR and IA for that slice. Finally, IA was expressed as a fraction of all ZAR by taking the sum of all infarcts and was reported as a percentage.

The left ventricle was immersion-fixed in 10% neutral formalin and embedded in paraffin wax (*n* = 5 for controls, *n* = 6 for exercised rats). Serial sections of 4-μm thicknesses were prepared. Apoptosis was evaluated via the terminal deoxynucleotidyl transferase-mediated dUTP nick-end labelling (TUNEL) method with the use of an *in situ* Cell Death Detection Kit, POD (1684817, Roche, Germany) according to manufacturer’s instructions, with some modifications.^10^

Briefly, the tissue sections were dewaxed and rehydrated by heating at 60°C, followed by washing in xylene and rehydration through a graded series of ethanol and double distillated water. Then the sections were incubated for 30 min at 21–37°C with proteinase K working solution (20 μg/ml in 10 mM Tris-Cl, pH 7.6). The sections were rinsed with PBS and incubated with the TUNEL reaction mixture for 1 h at 37°C in a humidified chamber.

As a positive control, sections were treated with DNase I (1 mg/ml, Sigma) for 10 min to introduce nicks in the genomic DNA. After converter peroxidase (POD) was added, the sections were incubated for 30 min at 37°C in a humidified chamber. Then the 3,3-diaminobenzidine substrate was added for the visualisation of nuclei with DNA nick-end labelling. The sections were counter-stained with toluidine blue to show normal nuclei.

The percentage of myocytes with DNA nick-end labelling was analysed by counting the cells exhibiting brown nuclei at × 40 magnification in five randomly chosen fields (1 mm^2^) in triplicate plates. The number of TUNEL-positive cardiomyocytes was counted by double-blinded observation.

## Statistical analysis

All statistical comparisons were made using SPSS 16.0 software (Chicago, IL) and were expressed as means ± SD. Work performed, pressures and flow data were analysed using repeated measures ANOVA. When a significant *p*-value was obtained, a *post hoc* Bonferroni test was used to determine the differences between the groups. Between-group comparisons of data of heart rate, infarct size, body weight, heart weight and apoptosis rate were made using the Student’s *t*-test. A *p*-value of < 0.05 was considered statistically significant.

## Results

Morphological data from the EXT and Sed rats are presented in Table 1. The rats in the EXT group had significantly lower body weights and higher heart weights than the Sed group (*p* < 0.05). In addition, heart-to-body weight ratio, as an index of heart hypertrophy, was greater in the EXT rats than the sedentary ones (*p* < 0.05).

**Table 1.** Effects Of Resistance Exercise On The Rat Morphology

	*Sed*	*EXT*
Body weight (g)	266 ± 13	259 ± 11
Heart weight (g)	0.75 ± 0.06	0.84 ± 0.06**
Body:heart ratio	2.8 ± 0.15	3.2 ± 0.18**

Values are mean ± SD (*n* = 10 rats); **p < 0.05, significantly different from the sedentary group; Sed: sedentary and EXT: exercise-trained rats.

**Fig. 1.**
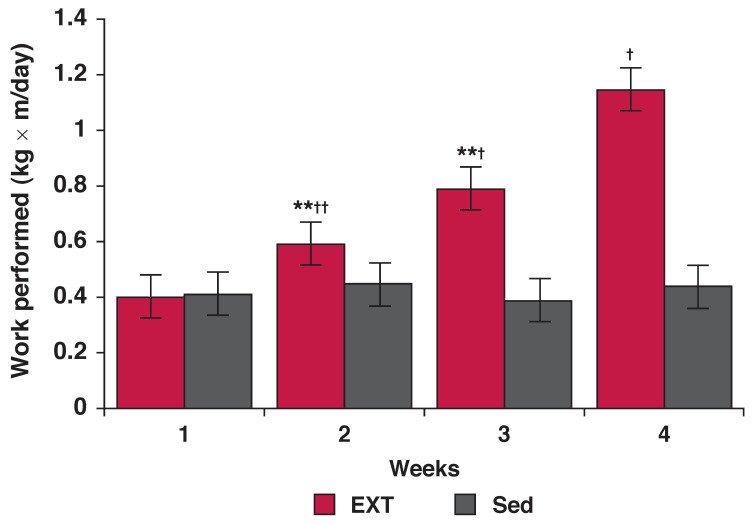
Work performed by rats after the end of each week of resistance exercise training. Values are mean ± SD (*n* = 20 rats); *p < 0.01, **p < 0.05 compared with previous week; ^†^*p* < 0.01, ^††^*p* < 0.05 compared with the sedentary group; Sed: sedentary and EXT: exercise-trained rats.

Fig. 1 shows a progressive increase in the weight-lifting ability of the EXT rats. Both the Sed and EXT groups had similar values for work performed in the first (week 1) of the protocol. The work performed at the end of weeks 2, 3 and 4 were significantly higher in the EXT rats than the Sed group (*p* < 0.05, *p* < 0.01 and *p* < 0.01, respectively) and their previous week’s values (*p* < 0.05, *p* < 0.05 and *p* < 0.01, respectively).

**Fig. 2.**
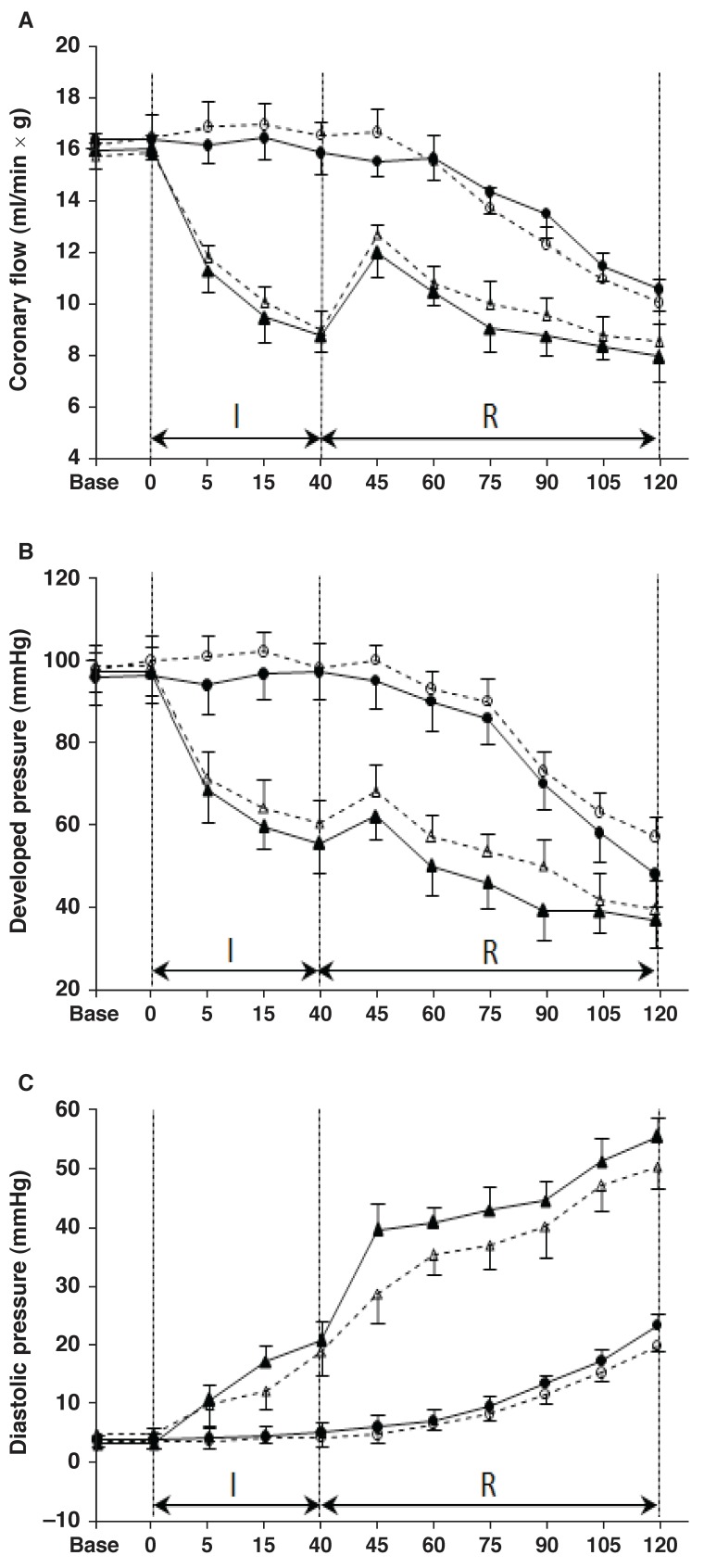
Haemodynamic indices of the heart during non-ischaemic time control (❍ exercised and ● sedentary rats; *n* = 6 for each), regional ischaemia (I) and subsequent reperfusion (R) (Δ exercised and ▲ sedentary rats; *n* = 12 for trained and *n* = 11 for sedentary animals). A: diastolic pressure. B: Left ventricular developed pressure (LVD P). C: Coronary flow. Values are mean ± SD.

Developed pressure, diastolic pressure and coronary flow changes during the time-control and ischaemia–reperfusion periods for the EXT and Sed groups are shown in Fig. 2. Baseline coronary flow, developed pressure and diastolic pressure were similar in the two groups. No between-group differences in developed or diastolic pressure were observed at any time point in the non-ischaemic time-control measurements. While diastolic pressure increased and developed pressure and coronary flow decreased in both the ischaemia and reperfusion periods (as indices of cardiac damage), there were no statistically significant differences between the EXT and Sed groups in these parameters.

**Fig. 3.**
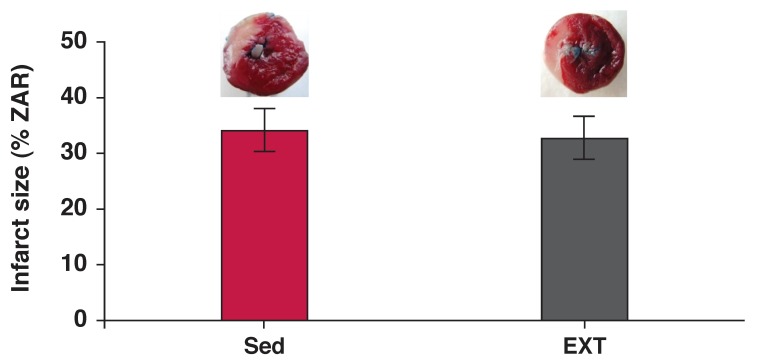
Effect of resistance exercise on the heart infarct size. Top: representative digital images of the stained heart. Non-necrotic viable tissue is dark, and infracted tissue is light. Bottom: quantification of average infarct size expressed as a percentage of ischaemic ZAR (zone at risk). Values are mean ± SD (*n* = 6 rats); Sed: sedentary and EXT: exercise-trained rats.

**Fig. 4.**
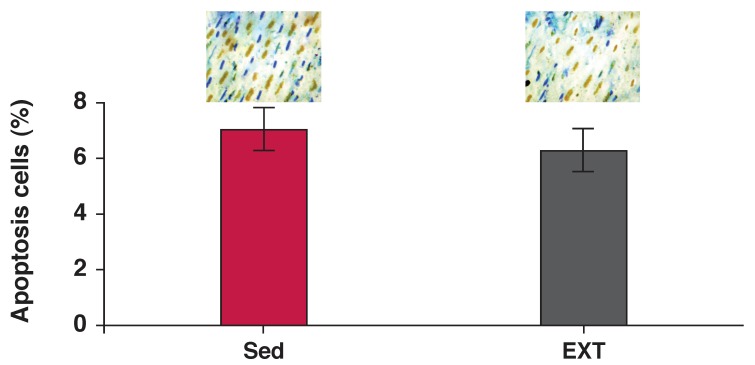
Effect of resistance exercise on the heart apoptosis rate. Top: cell death determined by the TUNEL method at × 40 magnification (black nuclei are the apoptotic cells). Bottom: comparison of apoptotic cell ratios in the two groups. Sed: sedentary and EXT: exercise trained rats. Values are mean ± SD (▲ = 6 for trained and ▲ = 5 for sedentary rats).

Figs 3 and 4 show the size of the infarction and the apoptosis rate, respectively in the hearts of the EXT and Sed groups. Resistance exercise training did not significantly change the infarct size or apoptosis rate.

## Discussion

Our previous study showed that 12-week resistance exercise training preserved the heart against IR-induced injury.^11^ Although there are some reports on the effect of resistance training on cardiac structure and function, to the best of our knowledge, this is the first study that has focused on the role of short-term resistance training in preserving the heart against IR-induced injury.

The main findings of the present study were that four weeks of resistance training: (1) increased the weight-lifting ability, (2) induced cardiac hypertrophy without any significant change in cardiac function, and (3) did not preserve the heart against IR-induced injuries, as evidenced by no change in the infarct size and apoptosis rate.

Weight loss, cardiac hypertrophy and work performed are some of the indices to characterise training efficiency. Previously, Barauna *et al*. reported that four weeks of resistance training in rats increased their weight-lifting ability and induced cardiac hypertrophy with no change in cardiac function.^4^ Progression in weight-lifting ability indicates training efficacy and development. While maximum heart rate or VO_2max_ are used to prescribe endurance exercise training,^5^ work performed may be a good indicator of resistance-training efficacy. Moritany and Vries showed that neuronal and muscular adaptations were involved in training-induced enhancement of the rat’s muscular strength.^12^

Resistance training is a known stimulus for cardiac hypertrophy due to pressure overload imposed on the heart during training.^13^ Our results are in agreement with pervious research.^4^,^13^ The precise underlying mechanism of resistance training-induced cardiac hypertrophy needs to be elucidated. In this regard it has been suggested that induction of angiotensin receptor type 1 (AT_1_) expression in the heart and elevation of circulating anabolic hormones may be involved.^13^,^14^

In this study, coronary flow, left ventricular developed pressure and diastolic pressure did not differ significantly between the trained and untrained rats. There are several published reports on the beneficial effect of resistance exercise on cardiac performance in patients with heart failure.^15^-^17^ In this regard it has been proposed that resistance training could improve stroke volume and ejection fraction without enhancement of cardiomegaly or cardiac deterioration.^15^-^17^

Few studies have investigated the effect of this type of exercise on cardiac function in healthy individuals and most did not report on changes in heart function after resistance training.^18^-^20^ Moreover, Barauna *et al*. reported that four weeks of resistance training did not change cardiac function in rats. Our results are in agreement with the results of these studies.^4^

Growing evidence indicates that IR-induced myocardial cell death is not limited to necrosis but also includes apoptotic cell death.^21^ For this reason, we measured ventricular apoptosis rate and infarct size in our study. The results show that short-term resistance training neither induces excessive damage to the heart nor preserves it against IR-induced injury, because apoptosis rate and infarct size did not change between our trained and control animal hearts.

While it has been shown that short- to long-term endurance exercise can protect the heart against IR-induced injury,^3^ some investigations did not report the beneficial effects of endurance exercise (up to 12 weeks) on cardiac performance, anti-oxidant defense and cell death rate.^10^,^22^ It has been proposed that these controversial results could have resulted from methological differences, such as type and duration of endurance exercise (swimming, treadmill or wheel running), time between the end of the training programme and sacrifice of the animals.^23^

## Conclusion

Previously we saw that 12-week resistance exercise training preserved the heart against IR-induced injury but the results of this study showed that four-week resistance training was unable to achieve this. Nevertheless, this is the first study with this purpose and a precise conclusion about this issue needs more investigation.

## References

[1] Hansen PR (1995). Myocardial reperfusion injury: Experimental evidence and clinical relevance.. Eur Heart J.

[2] Rao PR, Viswanath RK (2007). Cardioprotective activity of silymarin in ischemia-reperfusion-induced myocardial infarction in albino rats.. Exp Clin Cardiol.

[3] Powers SK, Quindry JC, Kavazis AN (2008). Exercise-induced cardioprotection against myocardial ischemia-reperfusion injury.. Free Radic Biol Med.

[4] Barauna VG, Rosa KT, Irigoyen MC, de Oliveira EM (2007). Effects of resistance training on ventricular function and hypertrophy in a rat model.. Clin Med Res.

[5] Zavorsky GS (2000). Evidence and possible mechanisms of altered maximum heart rate with endurance training and tapering.. Sports Med.

[6] Bjarnason-Wehrens B, Mayer-Berger W, Meister ER, Baum K, Hambrecht R, Gielen S (2004). Recommendations for resistance exercise in cardiac rehabilitation. Recommendations of the German Federation for Cardiovascular Prevention and Rehabilitation.. Eur J Cardiovasc Prev Rehabil.

[7] Tamaki T, Uchiyama S, Nakano S (1992). A weight-lifting exercise model for inducing hypertrophy in the hindlimb muscles of rats.. Med Sci Sports Exerc.

[8] Brown DA, Jew KN, Sparagna GC, Musch TI, Moore RL (2003). Exercise training preserves coronary flow and reduces infarct size after ischemiareperfusion in rat heart.. J Appl Physiol.

[9] Gao J, Fu W, Jin Z, Yu X (2006). A preliminary study on the cardioprotection of acupuncture pretreatment in rats with ischemia and reperfusion: involvement of cardiac β-adrenoceptors.. J Physiol Sci.

[10] Soufi FG, Farajnia S, Aslanabadi N, Ahmadiasl N, Alipour M, Alipour M (2008). Long-term exercise training affects age-induced changes in HSP70 and apoptosis in rat heart.. Gen Physiol Biophys.

[11] Soufi F G, Mahmoudi Saber M, Ghiassie R, Alipour M (2011). Role of 12-week resistance training in preserving the heart against ischemia-reperfusion-induced injury.. Cardiology.

[12] Moritani T, deVries HA (1979). Neural factors versus hypertrophy in the time course of muscle strength gain.. Am J Phys Med.

[13] Barauna VG, Magalhaes FC, Krieger JE, Oliveira EM (2008). AT1 receptor participates in the cardiac hypertrophy induced by resistance training in rats.. Am J Physiol Regul Integr Comp Physiol.

[14] Goto K, Takahashi K, Yamamoto M, Takamatsu K (2008). Hormone and Recovery Responses to Resistance Exercise with Slow Movement.. J Physiol Sci.

[15] Hambrecht R, Gielen S, Linke A, Fiehn E, Yu J, Walther C, Schoene N, Schuler G (2000). Effects of exercise training on left ventricular function and peripheral resistance in patients with chronic heart failure: A randomized trial.. J Am Med Assoc.

[16] Levinger I, Bronks R, Cody DV, Linton I, Davie A (2005). The effect of resistance training on left ventricular function and structure of patients with chronic heart failure.. Int J Cardiol.

[17] Palevo G, Keteyian SJ, Kang M, Caputo JL (2009). Resistance exercise training improves heart function and physical fitness in stable patients with heart failure.. J Cardiopulm Rehabil Prev.

[18] Colan SD, Sanders SP, Borow KM (1987). Physiologic hypertrophy: effects on left ventricular systolic mechanics in athletes.. J Am Coll Cardiol.

[19] Longhurst JC, Kelly AR, Gonyea WJ, Mitchell JH (1980). Echocardiographic left ventricular masses in distance runners and weight lifters.. J Appl Physiol.

[20] Pluim BM, Zwinderman AH, van der Laarse A, van der Wall EE (2000). The athlete’s heart. A meta-analysis of cardiac structure and function.. Circulation.

[21] Quindry J, French J, Hamilton K, Lee Y, Mehta JL, Powers S (2005). Exercise training provides cardioprotection against ischemia-reperfusion induced apoptosis in young and old animals.. Exp Gerontol.

[22] Morán M, Delgado J, González B, Manso R, Megías A (2004). Responses of rat myocardial antioxidant defenses and heat shock protein HSP72 induced by 12 and 24-week treadmill training.. Acta Physiol Scand.

[23] Ascensão A, Ferreira R, Magalhães J (2007). Exercise-induced cardioprotection – biochemical, morphological and functional evidence in whole tissue and isolated mitochondria.. Int J Cardiol.

